# A Rare Case of a Patient With Hairy Cell Leukemia Developing Blastoid Marginal Zone B-Cell Lymphoma

**DOI:** 10.7759/cureus.16239

**Published:** 2021-07-07

**Authors:** Yeshanew Teklie, Stephen Bell, Precious Idogun, Madhavi Venigalla

**Affiliations:** 1 Internal Medicine, Florida State University College of Medicine, Sarasota Memorial Hospital, Sarasota, USA; 2 Internal Medicine, Florida State University College of Medicine, Sarasota, USA; 3 Hematology and Oncology, Sarasota Memorial Hospital, Sarasota, USA

**Keywords:** hairy cell leukemia, blastoid marginal cell lymphoma, marginal zone lymphoma mzl, lymphoma, blastoid

## Abstract

There have been several case reports associating hairy cell leukemia (HCL) with non-Hodgkin’s lymphoma. However, it remains unclear whether these rare phenomena represent disease transformation or the coexistence of two separate disease entities. De novo marginal zone lymphoma can be routinely seen in elderly patients; however, in a patient with a history of HCL, a transformation is more likely than de novo blastoid marginal zone lymphoma. In this report, we present a rare case of a patient with HCL developing a high-grade blastoid marginal zone B-cell lymphoma (MZBCL).

## Introduction

Hairy cell leukemia (HCL) is an indolent, low-grade mature B-cell lymphoproliferative disorder characterized by splenomegaly, pancytopenia, and morphologically unique cells with characteristic cytoplasmic projections known as "hairy cells" [1-2]. HCL is a rare disorder and it accounts for only 2% of all cases of leukemia [2-3]. Its pathogenesis is thought to be related to mutations in the B-cell receptor (BCR). Diagnosis is made based on clinical presentation, flow cytometry (FC), laboratory data, and histological examination of the bone marrow (BM). Clinical presentation is usually related to cytopenia with patients having splenomegaly and or hepatomegaly due to extramedullary hematopoiesis with infiltration of marrow with neoplastic cells. Common presenting symptoms include significant anemia, which is seen in up to 85% of patients, thrombocytopenia seen in 60-80% of patients, and leukopenia seen in 60% of patients [4]. There have been several case reports associating HCL with non-Hodgkin's lymphoma [5], but the case we present here, one of aggressive blastoid non-Hodgkin's lymphoma, is very uncommon.

Initial workup for HCL includes complete blood count (CBC) with differentials, platelet count, and comprehensive metabolic panel as most patients present with pancytopenia. On the other hand, diagnostic workup includes peripheral blood (PB) smear review, BM biopsy, and immunophenotyping using immunohistochemistry (IHC) and/or FC [6]. The typical immunophenotype for HCL includes positive staining for CD11C, CD20, CD22, CD25, CD103, CD123, cyclin D1, and annexin A1 and negative staining for CD5 and CD10 [6].

On the other hand, the cases of blastic marginal zone lymphoma could be categorized into cases of de novo blastic marginal zone lymphoma and large-cell transformation arising in a background of a history of biopsy-proven marginal zone lymphoma. Primary cutaneous marginal zone lymphoma that undergoes blastoid transformation is rare and not well documented [7]. Clinical and pathological studies of eight cases have shown that cytogenetic abnormalities associated with blastoid transformation included deletion of chromosome 7q in all cases tested and expression of CD5 and CD23 [7].

## Case presentation

The patient was a 76-year-old male with a diagnosis of HCL 16 years before his current presentation. At that time, he had pancytopenia with a response to chemotherapy with cladribine and splenectomy. The patient had experienced a recurrence two years before the current presentation with FC showing CD103+ kappa-restricted lymphocytes, and a BM biopsy demonstrating low-burden hairy cells comprising 5% of cells with mild reticulin fibrosis. Due to the absence of cytopenia, a decision had been made to manage with active surveillance.

The patient presented to the emergency department for evaluation of fever, abdominal bloating, and rash. He denied any nausea, vomiting, fatigue, or changes in weight. He had no sick contacts, no recent travel, and had no pets at home. He had been evaluated at an emergency department three days prior to the admission where a CT abdomen-pelvis had shown large stool burden and nonspecific lymphadenopathy. He had been treated with polyethylene glycol and discharged home with instruction to follow up with his outpatient oncologist. He had been presumed to have newly refractory HCL and had been planning to restart cladribine; however, the patient had shown persistent symptoms and developed a pruritic, erythematous rash involving the chest, abdomen, and back, leading to the current hospitalization.

**Figure 1:**
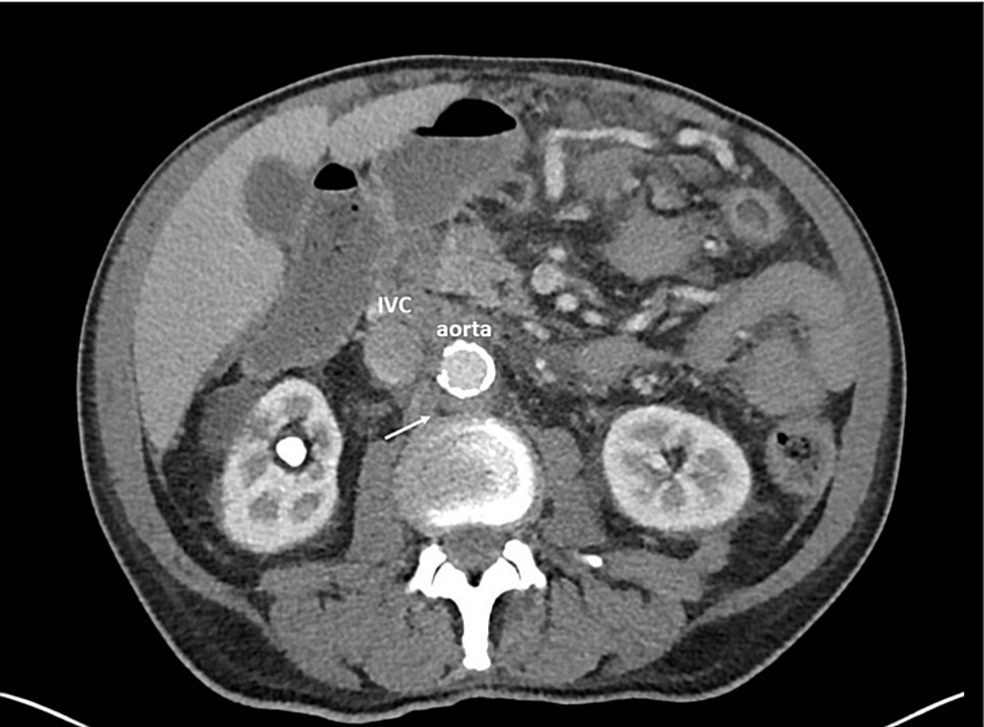
CT scan of the abdomen showing bulky paraaortic lymphadenopathy (white arrow) CT: computed tomography

**Figure 2:**
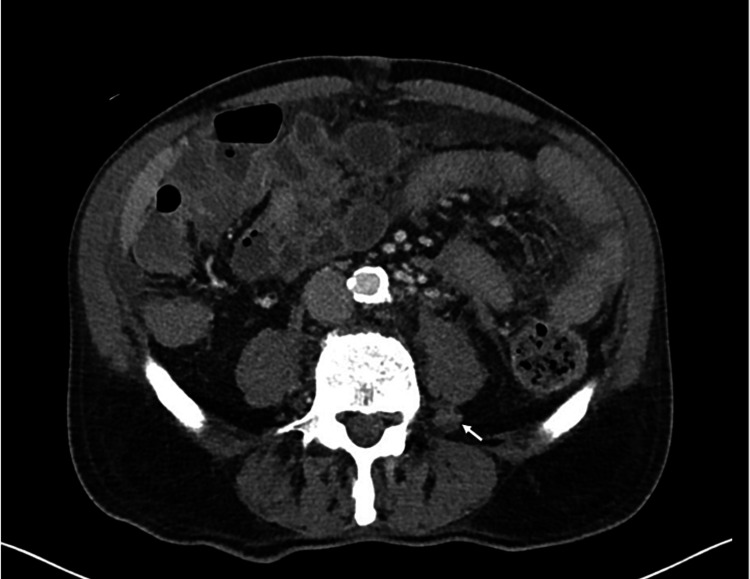
CT scan of the abdomen showing retroperitoneal lymphadenopathy (white arrow shows lymphadenopathy adjacent to the left psoas muscle) CT: computed tomography

The patient’s medical history was otherwise remarkable for coronary artery disease with prior myocardial infarction for which he had undergone percutaneous coronary intervention 10 years ago, hypertension, and hyperlipidemia. There had been no recent changes in his medications, which included 81 mg of aspirin daily, 75 mg of clopidogrel daily, 5 mg of lisinopril daily, and 40 mg of atorvastatin nightly. The patient had been born in the United States, had retired from working in the avionics industry, and was married, with one son. He was a former one-pack-per-day smoker for 30 years, having quit 30 years prior to the presentation. He did not drink alcohol or use illicit drugs. The patient denied any known family history of chronic diseases. On physical examination, the patient was found to be febrile with a temperature of 102 °F. Vital signs were otherwise within normal limits. The cardiopulmonary examination was unremarkable, but the abdominal exam was remarkable for mild diffuse tenderness with positive bowel sounds throughout. A diffuse maculopapular rash involving the chest, abdomen, and back was noted. A non-contrast CT of the thorax revealed diffuse lymphadenopathy involving the axillae, mediastinum, and hila with small bilateral pleural effusions and patchy ground-glass opacities bilaterally, as well as a ground-glass nodule in the right upper lobe measuring 2.5 cm. The spleen was surgically absent. A non-contrast CT of the abdomen-pelvis showed extensive bulky paracaval, paraaortic, mesenteric, and retroperitoneal lymphadenopathy with extrinsic compression of the pancreatic head and trace pelvic ascites (Figures 1, 2).

**Figure 3:**
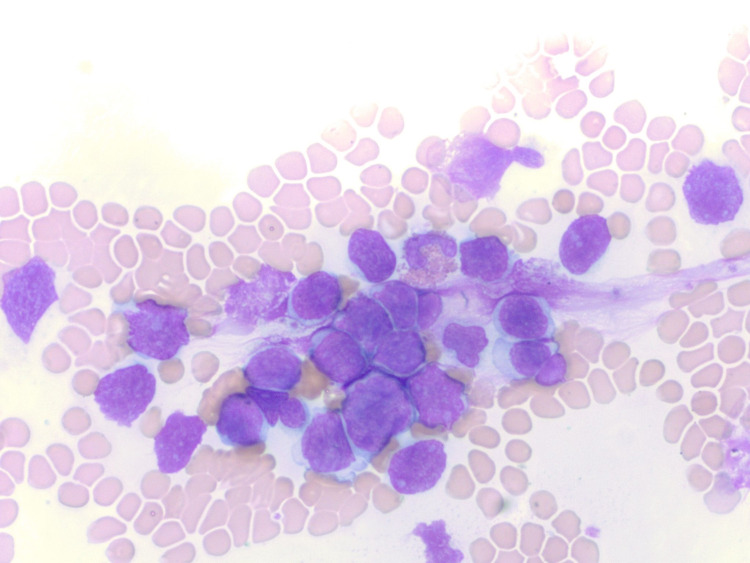
Clumps of large blastoid cells with prominent nucleoli* *Courtesy of Dr. Jeffrey Zacks

**Figure 4:**
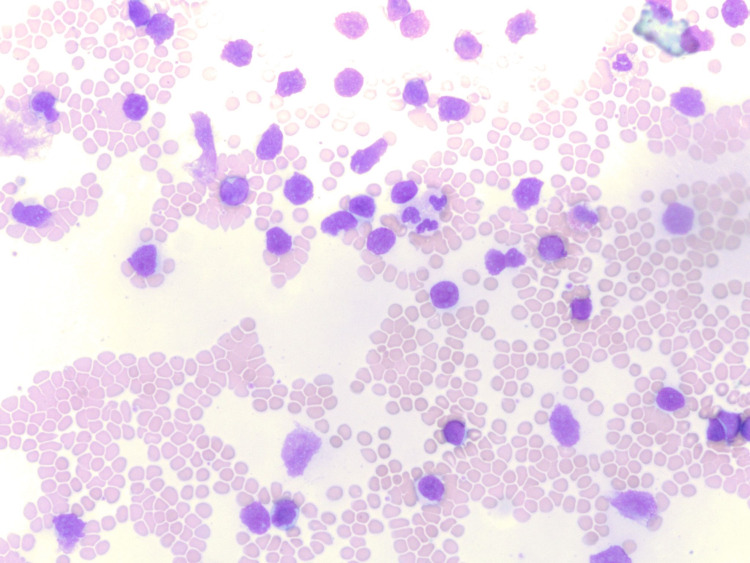
Large blastoid cells. Notice that the size of blastoid cells is about 2-3 times the size of normal RBCs* *Courtesy of Dr. Jeffrey Zacks

The patient’s laboratory testing was significant for leukocytosis of 128 x 10^3 ^u/L (normal range: 4.5-11 x 10^3 ^u/L) with 80% of cells being large blastoid lymphocytes with dense chromatin and large nucleoli on manual peripheral smear differential (Figures 3, 4). His chemistry testing showed hyponatremia of 131 mmol/L (135-145 mmol/L), creatinine of 1.66 mg/dl (0.7-1.30 mg/dl), lactate dehydrogenase of 476 U/L (87-241 U/L). The patient underwent CT-guided BM aspiration of the left ileum and ultrasound-guided right axillary lymph node (LN) biopsy. FC was performed on all specimens including PB with results as shown in Table 1. FC of the BM aspirate revealed an abnormal concentration of CD5+ B-cells, which stained bright for CD20, expressing monoclonal surface kappa light chain (kappa:lambda ratio: >100), as well as CD103, CD22, with subset CD11c. There was a variable expression of CD25. The neoplastic cells lacked CD23 and CD38. Lymphocytes comprised 68% of total cells, of which 96% were B lymphocytes. A similar pattern of IHC was identified in the LN, which was also demonstrating strong positivity for DBA-44. Staining of the BM and LN specimens with the Ki-67 antibody demonstrated a similar percentage of cells stained: approximately 20% in the former and 15% in the latter. The results suggested a similar proliferation profile in the two sites.

**Table 1:** Immunohistochemistry IHC: immunohistochemistry; PB: peripheral blood; LN: lymph node; BM: bone marrow; Pos: positive; Neg: negative; NT: not tested

IHC	PB	BM	LN
CD5	Pos	Pos	Pos
CD10	Neg	Neg	Neg
CD11C	Pos	Pos	Pos
CD19	Pos	Pos	Pos
CD20	Pos	Pos	Pos
CD22	NT	Pos	NT
CD23	Neg	Neg	Neg
CD25	NT	Pos/neg	NT
CD38	Neg	Neg	Neg
CD45	Pos	Pos	Pos
CD103	NT	Pos	Pos

Fluorescent in situ hybridization (FISH) studies on the PB demonstrated rearrangement of BCL6 but no rearrangement was identified in BCL2 or MYC. Additional FISH studies looking at common perturbations in chronic lymphocytic leukemia (CLL) were positive for trisomy 12 but negative for deletion of 6q, 11q (ATM gene locus), 13q, 17p (TP53 gene locus), or 11;14 translocation. There were additional chromosomal aberrations including rearrangements involving chromosomes 6, 11, 12, 13, 14, and 17, which are nonspecific and consistent with a complex karyotype. Further IHC testing on the BM specimen was positive for PAX5 but negative for SOX11 and BCL1. Cytogenetic testing revealed near tetraploid karyotype (84~85, XXY) with multiple perturbations including deletion in chromosomes 1, 2, 5, 9, and 17, and duplication in chromosome 6 consistent with a complex karyotype. Complex near tetraploid karyotype is associated with an aggressive disease with a poor prognosis. BRAF mutation was not detected. The patient was diagnosed with CD5-positive B-cell lymphoproliferative disorder with IHC, BM and FC features suggestive of blastoid marginal zone B-cell lymphoma (MZBCL).

## Discussion

The presence of greater than 80% of large blastoid cells with prominent nucleoli and a high nucleus-to-cytoplasm ratio on the peripheral smear makes the patient’s pathology an aggressive neoplasm. Diagnosis of blastoid marginal cell lymphoma is a diagnostic and therapeutic challenge, especially when it is a transformation from a more indolent neoplasm such as HCL. No specific genetic or IHC has been identified, to date, to diagnose marginal zone lymphoma, and specific diagnosis is based on the integration of clinical features with diagnostic workup as in our case. Chemotherapy was subsequently initiated with rituximab and bendamustine. This case presentation likely shows a rare transformation of HCL into an aggressive blastoid marginal cell lymphoma with significant leukocytosis and extensive lymphadenopathy in a patient presenting with fever, rash, and abdominal bloating/discomfort due to high tumor burden. Although HCL is a neoplasm of indolent mature B lymphocytes, it can transform into an aggressive neoplasm including blastoid marginal cell lymphoma.

## Conclusions

Patients with HCL should be closely followed up with periodic laboratory testing including CBC, peripheral smear review, and imaging studies including CT scan and PET scan to assess for any leukocytosis, pancytopenia, abnormal morphology, or lymphadenopathy. Patients with HCL, therefore, should be followed up with frequent monitoring of CBC at least every six months and clinical investigation at least every year. Another important lesson from this case is the fact that cells that mimic blasts on PB smear are not always blasts that are normally consistent with acute lymphoblastic leukemia or any acute leukemia. Hence, the patient’s clinical presentation together with BM biopsy results and immunohistology/cytology and cytogenetic results have to be reviewed to come up with an accurate diagnosis. The differential diagnosis for blasts or pseudoblasts on peripheral smear, hence, should include acute leukemias as well as blastoid lymphomas, including marginal cell lymphoma. Previous case reports only mention the association of HCL with high-grade lymphoma but the focus on the blastoid component of high-grade lymphoma is less prominent, making this case presentation unique. Besides, this case had complex immunohistochemical and chromosomal abnormalities, unlike previous similar case reports.
